# Linking Animal Welfare and Antibiotic Use in Pig Farming—A Review

**DOI:** 10.3390/ani12020216

**Published:** 2022-01-17

**Authors:** Rita Albernaz-Gonçalves, Gabriela Olmos Antillón, Maria José Hötzel

**Affiliations:** 1 Campus Santa Rosa do Sul, Instituto Federal Catarinense, Santa Rosa do Sul 88965-000, SC, Brazil; rita.silva@ifc.edu.br; 2 Laboratório de Etologia Aplicada e Bem-Estar Animal, Universidade Federal de Santa Catarina, Rod. Admar Gonzaga 1346, Itacorubi, Florianópolis 88034-001, SC, Brazil; 3 Department of Clinical Sciences, Swedish University of Agricultural Sciences, 75007 Uppsala, Sweden; gabriela.olmosantillon@gmail.com

**Keywords:** antimicrobial use, antimicrobial resistance, intensive farming, stress disease model, sustainability

## Abstract

**Simple Summary:**

Minimising stress in intensive pig farms is paramount to raising immunocompetent pigs. This entails providing the pigs with living conditions (from birth to the point of slaughter) free of pain, stress, and suffering and simultaneously providing conditions that generate positive affective states. Our review aims to study the relationship between chronic stress, illnesses, their impact on antibiotic use (AMU), and potential housing and management improvements to tackle stress and AMU. According to the literature, pigs kept in crowded, barren conditions, with poor microclimatic conditions, and subject to painful and stressful weaning practices present redirected behaviours, poor immune-competence, and weaker bodies. In turn, pigs are more vulnerable to circulating pathogens and severe secondary infections, which is conducive to high AMU for the sake of the animals’ health. Simultaneously, we compiled a list of possible solutions for the current poor environment and practices, including a call for the pig industry to broaden its concept of animal welfare beyond the current biological/productivist scope. We propose that advocating for an industry with enhanced animal welfare is a crucial response to the international call to combat antimicrobial resistance and the social demand for ethically sustainable animal production.

**Abstract:**

Preventative measures, such as biosecurity and vaccinations, are essential but not sufficient to ensure high standards of health in pig production systems. Restrictive, barren housing and many widely used management practices that cause pain and stress predispose high-performance pigs reared in intensive systems to disease. In this context, antibiotics are used as part of the infrastructure that sustains health and high levels of production in pig farms. Antimicrobial resistance (AMR) is a global emergency affecting human and animal health, and the use of antibiotics (AMU) in intensive livestock farming is considered an important risk factor for the emergence and spread of resistant bacteria from animals to humans. Tackling the issue of AMR demands profound changes in AMU, e.g., reducing their use for prophylaxis and ending it for growth promotion. In support of such recommendations, we revise the link between animal welfare and AMU and argue that it is crucial to sustainably reduce AMU while ensuring that pigs can live happy lives. In support of such recommendations, we aimed to revise the link between animal welfare and AMU in pigs by analysing stress factors related to housing and management and their impact on pig welfare. In particular, we reviewed critical management practices that increase stress and, therefore, pigs’ susceptibility to disease and reduce the quality of life of pigs. We also reviewed some alternatives that can be adopted in pig farms to improve animal welfare and that go beyond the reduction in stress. By minimising environmental and management stressors, pigs can become more immunocompetent and prepared to overcome pathogenic challenges. This outcome can contribute to reducing AMU and the risk of AMR while simultaneously improving the quality of life of pigs and, ultimately, maintaining the pig industry’s social license.

## 1. Introduction

Animal welfare, together with environmental issues, is one of the biggest challenges of agriculture in the 21st century [1]. Significant advances in the scientific and legal recognition of animal sentience and welfare have been achieved in the past few decades that guide this discussion. The OIE encompasses the basic elements that constitute animal welfare in its Terrestrial Animal Health Code, stating that “an animal experiences good welfare if the animal is healthy, comfortable, well-nourished, safe, is not suffering from unpleasant states, such as pain, fear, and distress, and is able to express behaviours that are important for its physical and mental state” [2]. However, intensive pig production systems, in general, fail many of these goals and offer few conditions for animals to experience positive affective states. As a result, many pigs spend their entire lives in conditions that do not ensure “a life worth living” [3]. Confinement housing limits free movement, the expression of highly motivated natural behaviours, such as nesting and rooting, and socialisation; additionally, pigs are exposed to painful management practices from moments after birth [4,5]. As a result, the pigs are stressed and more vulnerable immunologically and predisposed to contract environmental pathogens [6]. In addition, high stocking densities contribute to the spread of respiratory and enteric diseases. Altogether these factors contribute to the use of antibiotics (AMU) to control and prevent infectious outbreaks on farms [7,8]. The global consumption of veterinary antimicrobials is projected to increase 11.5% by 2030, over the estimated 93,000 tonnes used in 2017, when 10 countries used 75% of all veterinary antibiotics used in animal production (China = 45%; Brazil = 7.9%; the United States, Thailand, India, Iran, Spain, Russia, Mexico, and Argentina) [9]. Several countries have banned the use of antibiotic growth promoters and have limited active principles for human use only [10]. However, given the high use of antibiotics for disease prevention, this ban may be insufficient to reduce AMU [11]. Legislation restricting AMU in livestock production has changed attitudes and practices towards non-therapeutic AMU, most notably in the EU and particularly in some countries (see for example [8]), but the use of veterinary antibiotics in the world is still quite expressive. For example, in a survey of pig herds in Germany, the authors identified that pigs with an expectation of 200 days of life received antimicrobials for 48.5 days [12]. A Brazilian study identified that pigs received an average of 7 different antibiotic active principles during 73.7% of their life [13].

The contribution of AMU in intensive pig farming to the emergence of antibiotic-resistant bacteria in humans raises important ethical, social, and public health concerns [14,15]. Resistance is a spontaneous process of bacteria that can be accelerated by the inappropriate use of antibiotics [16]. Resistance genes can be spread by vertical transmission, where the original bacterial cell transmits the resistance determinant to the offspring, or via horizontal, inter and intra-species transmission [17]. The potential for dissemination is greatest through horizontal transmission due to the mobile genetic elements that can spread in the environment and be incorporated by other bacteria, making animals highly relevant antibiotic resistance (AMR) agents [16,17]. AMR can be spread via direct or indirect contact between humans and farm animals or biological substances or via the consumption of contaminated food products; additionally, veterinarians, farmers, abattoir workers, and food handlers and their families may be an entry route of resistant bacteria into the community and health care settings [18]. The transfer of multi-resistant bacteria between animals and humans is a critical concern, considered by health agencies as a worldwide public health emergency [19]. For this reason, international leaders have called for an urgent reduction in AMU in livestock and the development of sustainable food systems [20].

To promote strategies for prudent AMU and minimise AMR, the FAO, WHO, and OIE have created a three-pronged approach—One Health—that links aspects of human, animal, and environmental health into transdisciplinary public policies. One Health is a holistic health concept that proposes viewing human and animal health as interdependent and simultaneously connected with each other and with the ecosystems in which they coexist in a balanced relationship [21]. The One Welfare concept is based on the understanding that good animal welfare can reflect upon humans and the environment by ensuring food safety, improving human health, environmental sustainability, worker safety, rural development, gender equality, and social justice [22]. The aims of this review are to uncover the relationship between animal stress and health; to identify the main stressors in specific periods of the life of pigs reared in intensive systems and discuss how they affect pig welfare and health; and, finally, to point out some interventions that can improve the welfare and health of pigs and, potentially, contribute to the goal of reducing AMU in pig farming.

## 2. Stress as a Trigger for Disease

Understanding the physiological processes underlying the stress response and how they influence the pig’s immune system and health can shed light on the relationship between pig welfare and AMU. Therefore, we will define some terms that are relevant for this review.

The stability of life-sustaining physiological parameters (pH, oxygenation, temperature, blood glucose) is referred to as homeostasis [23,24]. When an individual’s homeostasis is threatened in some way, e.g., by environmentally adverse situations, the homeostasis imbalances are compensated through a process called allostasis, which mobilises resources, such as energy, for the short-term adaptation/acclimatisation of the organism [23]. Evolutionary mechanisms of allostasis trigger the stress response through the autonomic nervous system and the hypothalamus–pituitary–adrenal axis (HPA). This prepares an alarm response, which is activated by the release of adrenocorticotropic hormone (ACTH), which, in turn, stimulates cortisol secretion, with metabolic, cardiovascular, and immunological effects. The excessive activation of allostatic mechanisms interferes with the basic energy mechanisms that maintain growth, reproduction, and the immune system, resulting in harmful stress (herein referred to as distress) that causes suffering and reduces animal welfare [6,25]. According to Moberg and Mench [6], the stress response can be divided into three stages: the recognition of a stressor, the biological protection against that stressor, and the consequences of the stress response. The consequences of the stress response will determine whether the animal is undergoing a temporary negative experience (i.e., acute stress) or suffering from chronic stress, the latter having the most damaging effects on the animal’s health and welfare [6].

Psychological stressors (e.g., distress, frustration, boredom) may be as damaging to the immune system as pathogenic aggressors. The perception of a threat stimulus by the central nervous system triggers a biological defence that activates four types of responses: a behavioural response, an autonomic nervous system response, a neuroendocrine response, and an immune response [6]. Abnormal behaviours in pigs (e.g., belly-nosing, tail and ear biting, aggressiveness) are examples of behavioural responses to chronic stress [25].

Fear plays an important role in helping animals cope with environmental stressors by motivating them to avoid potentially dangerous situations [26]. Fear influences animal performance and welfare via a classical stress response that involves physiological responses that aim to provide energy for immediate use by the body in preparation to flee or face aggression [26]. The reduction in staff/animals derived from intensification and the restriction of contact with humans with the advent of automated systems have contributed to more aversive reactions in routine management, poor handling, and negative interactions [27]. The intensification of livestock production also added problems, such as unskilled, often unmotivated workers due to high turnover, low pay, and lack of sufficient training [28,29]. Therefore, negative human–animal interactions at the farm may contribute to increased susceptibility to disease by activating energy-costly stress responses, especially when these interactions are persistent or frequent.

According to the resource allocation theory, the animal metabolism will always spend the least amount of resources, selecting which metabolic function to benefit through a partition of metabolic resources; when certain resources are consumed by a given metabolic function, they are not available for other functions [30]. Pigs that suffer from chronic stress have a reduced natural ability to mount a successful response to an immune challenge [31]. Additionally, pig strains genetically selected for high production performance are more susceptible to disease because they allocate metabolic resources to meet physiological demands at the expense of the immune system [32]. For example, pigs that need to spend more metabolic resources for growth will have fewer nutrients available for the immune system, increasing their vulnerability to disease. Likewise, when the protein synthesis necessary for rapid growth, reproduction, and immune processes is depressed by chronic stress, energy reserves are mobilised [6]. In normal situations, this response favours the survival of the individual; however, chronic stress can be harmful to the organism due to its continuing nature.

## 3. Sources of Stress in Pig Farm Management

**Figure 1:**
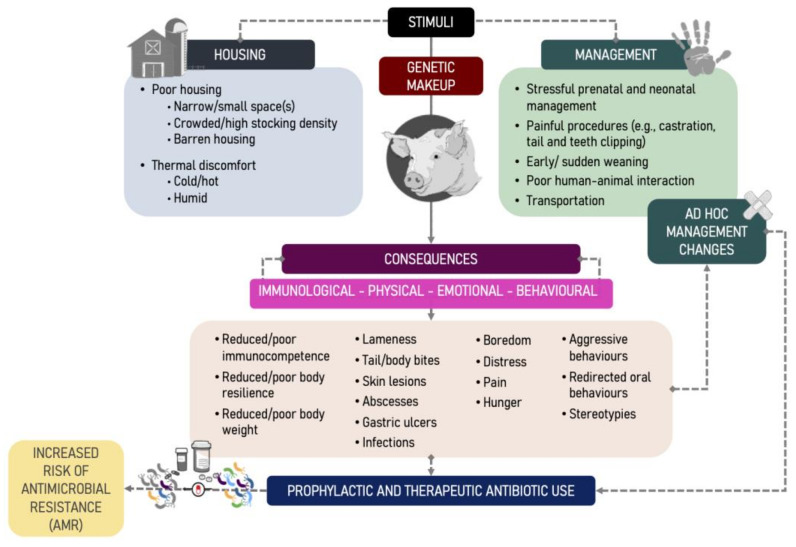
The biological pathway on how human-modulated stressors that challenge pigs’ immunological, physical, emotional, and behavioural status act as risk factors (direct/indirect) for antibiotic use (AMU) and antibiotic resistance (AMR) on farms. Lines indicate direct/indirect association, not the strength of association or causation. The observed AMU by the pig industry will be the sum of stressors and related management decision-making to proactively or reactively (e.g., ad hoc solutions) deal with such stressors. Literature and practical knowledge indicate that ad hoc management changes (e.g., cross-fostering or prophylactic antibiotic treatments) are quick fixes rather than rooted modifications. Thus, these management practices are equally risk factors of AMU/AMR in pigs.

The concepts and physiological mechanisms that we have reviewed shed light on the mechanisms that make pigs exposed to multiple stressors more vulnerable to disease. In this section, we will address some of the main stressors that challenge pigs reared in intensive conditions, dividing them into two types: those derived from the housing environment and those generated by management practices typically adopted in intensive pig farming (Figure 1). The first type is related to the physical limitations imposed by intensive housing, such as restriction to movement, socialisation and expression of natural behaviours, and unfavourable climatic conditions, which can cause discomfort, injuries, lameness, and abnormal and stereotyped behaviours. The second covers sow feeding management, prenatal stress, neonate management, weaning, early transport, mixing unfamiliar animals, mutilations, and human–animal interactions. When relevant, we point to the relationship between these stressors, disease, and AMU.

### 3.1. Housing Stressors

#### 3.1.1. Housing That Limits the Ability of Pigs to Move and Express Natural Behaviours

Intensive pig production models have been designed to raise as many animals as possible in small spaces and with short production cycles. In these systems, high stocking density often goes hand in hand with barren environments. Pigs are reared in barren housing environments in the breeding, weaning and fattening phases. Monotonous environments generate boredom, a negative emotional state [33]. High stocking densities exacerbate the problems of barren environments, resulting in aggression and redirected behaviours performed on conspecifics [34].

The frustration associated with lack of environmental stimuli and the inability to root can manifest in behaviours such as tail and ear biting of pen mates [35], a major welfare problem in growing and fattening pigs and an important source of severe infections and abscesses [36,37]. Damage from tail biting can range from a bite mark to a serious injury and, in more severe cases, it can cause the bitten pig to die [37] and is a reason for AMU [36,38]. Partial tail docking of piglets at birth is the most commonly used practice to prevent tail biting [37]. Nevertheless, it has been shown that tail docking is not sufficient to eliminate biting behaviour when pigs are challenged by a stressful environment [37], and in some cases, when piglets have the tail docked, the behaviours may be redirected to other parts of the body [39].

Individual confinement is used to house breeding sows, in many countries for their entire productive life. Gestating and lactating sows and boars are housed in barren crates where they cannot walk or turn around and their ability to perform highly motivated behaviours is restricted [40,41]. This housing facilitates feeding and management and optimises space use, but given that it limits the foraging behaviour, it causes chronic hunger and stress in gestating sows, which is expressed in abnormal and stereotypic behaviours [41]. Gestation crates have been increasingly banned in some countries but continue to be used in many parts of the world [42]. Farrowing crates, designed to facilitate management and to minimise piglet crushing, are the most common housing for farrowing and lactating sows in commercial farms in most countries [43]. Farrowing crates deprive sows of nesting, a highly motivated natural behaviour that has not been changed in modern pig lines genetically selected for productive traits compared to their predecessors [44]. Sows confined in farrowing crates show restlessness, frequent changes in body position, intermittent grunts, grinding teeth, and bar biting and biting other parts of the crates [45]; this housing can also generate frustration and aggressiveness in pre-parturient sows [46].

The lack of movement resulting from permanent confinement has several health consequences. It can lead to poor cardiovascular function and bone and muscle weakness; in heavy pigs, it can also predispose to locomotor disorders, such as lameness [47]. In sows, lameness is an important predisposing factor for urinary tract infections. As pregnancy progresses, the sows become heavier and may have difficulty moving because of the pain, which predisposes them to remain in the sitting dog position for longer periods and reduce water consumption; this often leads to infrequent urination [48,49], which, together with faecal contamination of the perineal region, predispose sows to bacterial urinary infections [48], reported as the main cause of prophylactic AMU in pregnant sows [7,50]. These infections predispose animals to reproductive disorders, such as reduced litter size, return to oestrus, abortion, anoestrus, and postpartum dysgalactia syndrome [48], which also lead to increased AMU [51].

#### 3.1.2. Housing That Causes Thermal Stress

The thermal comfort zone, i.e., the ambient temperature range in which the thermoregulatory effort is minimal, varies among individual pigs according to the amount of endogenous heat produced and the environmental conditions. Selection for lean tissue changed the proportion between body protein and fat, with protein accumulation generating more body heat, increasing pigs’ susceptibility to variations in temperature [52]. The general physiological response to thermal stress is similar to the chronic stress response discussed earlier in this review [6]. Two harmful physiological mechanisms result from thermal stress: the first mechanism is associated with a hormonal imbalance; the second mechanism involves an increase in pro-inflammatory cytokines that compromise intestinal integrity, alter immune function, and predispose pigs to infections [53,54]. Heat-stressed pigs are more prone to enteritis. To dissipate body heat, blood flow from the viscera to the periphery deviates, causing intestinal hypoxia, ATP depletion, and oxidative stress of enterocytes [53]; this also destabilises the intestinal barrier, making it permeable to Gram-negative bacteria and other disease-causing antigens [55,56]. Under low temperatures, ammonia concentrations within the housing facilities increase, which causes irritation and changes in the respiratory mucosa and leads to respiratory infections [57], one of the main causes of AMU [38]. Exposure to both cold and heat is an important stressor associated with the transport of weaned piglets. While high temperatures during travel may increase the risk of dehydration [58,59], cold-exposed piglets enter hypothermia and take time to recover when they reach their destination [58]. Moreover, thermal variations during travel can increase the stress level of piglets and affect their postweaning recovery [59].

### 3.2. Common Management Practices as Stressors

#### 3.2.1. Feeding Strategies as a Source of Stress

Feed restriction in pigs is a management practice widely used to limit weight gain in breeding sows or for compensatory gain purposes in fattening pigs. Sows’ diets are designed to limit caloric intake and excessive weight gain during gestation [60]. Because they are usually fed twice a day and consume the low-fibre concentrated food quickly, sows remain hungry and highly motivated to seek food [61]. Chronic hunger stress can induce the expression of redirected and stereotyped oral behaviours [60]. In many cases, sows are housed in gestation crates, which adds to the stress caused by food deprivation; unable to forage, stressed sows bite cage parts, exhibit sham-chewing, and smell and lick the floor and other parts of their cages excessively [61]. In group housing, deficiencies in feed management can lead to social stress and an increase in competition for feed, resulting in agonistic interactions, injuries to the vulva and tail biting [60,61].

Compensatory growth is a physiological phenomenon that occurs when an animal that has undergone a period of nutritional stress is fed ad libitum. Feed restriction during the growing and fattening periods is conducted by reducing the amount of feed or specific nutrients in the diet, as a way to decrease fat in pig carcasses at slaughter, or to stimulate compensatory weight gain in low-birth-weight piglets, improving feed conversion and reducing feed costs [62]. Providing low amounts of food or nutrients exposes pigs to unnecessary stress. The impact of these practices on pigs’ welfare needs to be investigated, e.g., the potential for hunger to increase activity, aggression, and tail biting.

Lastly, long fasting periods and chronic hunger, together with environmental thermal variations and disease outbreaks, may lead to gastric ulcerations [63,64]. The prevalence of gastric ulcers at slaughterhouses can vary between 32% and 65% [64], but this figure may be underestimated, as ulcers are often subclinical. Other risk factors for gastric ulcerations include feed particle size, gastric microbiota composition, hormonal changes, *Helicobacer suis* infections, and low birth weight [64,65,66].

#### 3.2.2. Early Life Management

##### Prenatal Stress

The gestation environment may have repercussions on piglet welfare and health, given that prenatal stress in sows can impair growth and modify immune function, stress reactivity, and the behaviour of the offspring [67,68]. Piglets born to sows stressed during gestation have reduced immune capacity and are more susceptible to infections during the lactation and pre-weaning periods [67]. Stress in gestating sows results in high levels of glucocorticoids that cross the placental barrier and may affect the foetal HPA axis maturation through hippocampal cell death and loss of cognitive functions [69]. Prenatal stress can also alter the offspring phenotype, e.g., the daughters of stressed sows are more anxious, restless during farrowing, more reactive, and bite their piglets more [69].

##### Neonatal Management

Nursing is a critical period for the survival of piglets when colostrum is the main source of antibodies, heat, and energy for the newborn [70]. The supply of colostrum in the first hours of life is essential for intestinal protection and passive immunity to piglets [71]. Piglets of different weights, typical of large litters, do not receive the same amount of colostrum. Weak or small piglets are especially susceptible to neonatal diarrhoea [72,73]. The main forms of diarrhoea control are vaccines and antibiotics, in particular penicillin and macrolides [12]. In Brazil, the use of injectable antibiotics in newborn piglets to prevent neonatal diarrhoea and other infections is commonly reported [13,50]. However, this practice may have deleterious effects on the intestinal microflora and the immune system of piglets, as it has been shown that early AMU can have a programming effect on the immune system. Gut dysbiosis in early life can promote an exacerbated inflammatory reaction, increasing the risk of colitis and impairing the immune response throughout life [74,75].

##### Cross-Fostering and Artificial Rearing

Cross-fostering and artificial rearing are two practices used to help small and slow-growing piglets gain weight. Cross-fostering consists of separating the newborn piglets by weight and distributing them in nursing sows according to piglet size and sow’s milk production, i.e., to equalise litters [76,77]. Artificial rearing is conducted with automatic systems that provide heat and feed to 2 to 14-day-old piglets [78]. Additionally, in some farms, piglets may be weaned 7 to 21 days after littermates and maintained with a nursing dam or nursed artificially, mixed with younger piglets of a similar weight [71], posing a disease risk to the younger pigs they get mixed with [79]. Hyperprolific sows, increasingly used in intensive farms, generate more viable eggs; however, the limitations of intrauterine space increase foetal competition, which is reflected in a greater number and proportion of piglets with low birth weight [80], which exacerbates the use of cross-fostering and artificial rearing practices [81]. However, cross-fostering and artificial rearing may have negative impacts on the health and welfare of piglets [78,79].

During lactation, the sow transfers microorganisms to piglets, which is essential to the establishment of permanent intestinal and respiratory microbiomes that will assist in the maturation of the piglets’ immune system [82,83]. Piglets deprived of maternal contact and reared artificially present reduced pulmonary development, respiratory immune response and microbiome of the lungs [82,84].

The recommendation is that cross-fostering is performed between 14 and 24 h postpartum to allow colostrum ingestion by piglets and the establishment of the teat order. Piglets transferred between 2 and 7 days postpartum or those transferred into groups with older piglets have difficulty integrating and exhibit more ambulation and vocalisations, taking a longer time to suckle compared to piglets transferred to younger foster litters [77]. Newborn piglets have not yet received maternal antibodies, and by mixing them with older piglets, they are exposed to pathogens to which they are not protected, increasing the risk of infection [85]. Early cross-fostering may be also harmful to piglets because colostrum production and the concentration of colostral immunoglobulins reach their peak within 14 h postpartum; thus, piglets that are fostered before this time will not have received sufficient colostrum, which makes them more susceptible to environmental pathogens [86]. However, in practice, cross-fostering is conducted after 7 days postpartum, or even later, and sometimes multiple times (e.g., [76,87]). The potential for transmission of pathogens in late cross-fostering is highly relevant to this discussion. Additionally, with each group change, the teat order needs to be re-established, which can be stressful and have detrimental effects on piglet survival, growth, and behaviour [71]. Growth may be impaired in adopted piglets due to fights over teats and shorter feeding bouts [88].

##### Weaning Stress

Weaning is one of the greatest stressors in a piglet’s life [89] and one of the main risk factors for diarrhoea in the postweaning period [73], which contributes to a large proportion of AMU in pig farms (e.g., [50,90,91,92,93]). The time the piglets stay with their mother has important physiological and psychological effects on the piglets’ development. Under natural conditions, the piglets are gradually weaned, completely separating from their mother between 17 and 20 weeks of age [94]. Under typical commercial conditions, this process is conducted abruptly, between 3 and 5 weeks of age. Such a strategy, widely practised in intensive farms, eliminates or shortens important stages of the physiological and emotional development of piglets [89]. Early weaned piglets are subjected to the simultaneous social and psychological stress caused by losing milk, being socially separated from the mother and the siblings, and moved to a new environment, where they are usually mixed with unfamiliar animals and often transported between farms [87,89]. Biting, nosing, and abnormal behaviours are common among weanling piglets and constitute redirected sucking behaviours associated with early weaning, barren environments and hunger, and possibly have a genetic component; these behaviours can result in skin lesions on the recipient’s belly and flank, causing skin injuries, pain, and difficulty resting [95].

From an emotional point of view, the early negative experience of maternal separation has effects on the hippocampus, so early weaned piglets have behavioural and cognitive impairment [96]. Sick and feverish piglets, in an attempt to conserve body energy, reduce their activity and remain lying down without feeding; however, weaning management prevents piglets from adopting such energy conservation strategies [89], making them more vulnerable to infections. Psychological and physical stressors associated with weaning imply energy expenditure and reduced food consumption, resulting in body weight loss in the first week after weaning [73,97]. All these factors, added to the lack of familiarity with solid feed, can lead to transient anorexia, intestinal inflammation, gut microbiota disorders, and behavioural disorders that result in a high occurrence of diarrhoea [73].

##### Transportation of Young Pigs

With the growing trend of pig production being conducted at specialised breeding sites, piglets may be transported for many hours after weaning to their destination at fattening units [87]. Every year millions of weaned piglets as young as 17 days of age are transported over long distances. The way this transport is carried out can have a significant impact on the welfare of these piglets [98]. There is a knowledge gap regarding this topic, as the scientific literature refers more often to the transport of pigs at slaughter age. However, it is understood that the stress factors during transport are the same in young and adult pigs, with the aggravation of the frailty of younger animals and the concurrent weaning stress. Transport stress is acute and may be followed by dehydration and protein catabolism [99]. Some stressors associated with transportation are temperature variation, the mixture of unknown animals’ hunger and thirst, loading and unloading, vibration, and noise [59]. Travel time affects the welfare of piglets during and after transportation [100]. For example, piglets transported for long journeys between 12 and 24 h are more prone to dehydration [98]. Transport speed can also be detrimental to the piglets; with fast motion, the piglets often lie down and stand up, indicating imbalance and vulnerability to falls [99]. Fasting associated with transport also causes deleterious effects on the health of weaning piglets. Pigs can lose about 4% of their body weight fasting for 18 to 24 h, causing catabolism of body reserves over 24 h [99,100,101]. Importantly, transport can be a pathogen carrier. Reducing vertical disease transfer, increasing productivity and overall efficiency of the farm are some justifications for the practice of site segregation of piglets [59]. However, the mixture of piglets from various origins is a source of pathogen transfer. Additionally, another epidemiological aspect to be considered is the emission of contaminating particles and the spread of resistant bacteria during transport [99]. The transmission of disease through transport and mixture of piglets of different origins and the impact on AMU and AMR are important knowledge gaps.

#### 3.2.3. Painful Procedures and Parturition as Sources of Pain in Pigs

Most piglets undergo several painful management practices during the first days after birth, mostly without any use of pharmacological tools to avoid or reduce the pain [5]. The main painful practices are teeth clipping or resection, tail docking, ear notching for identification, iron injection, and castration of male pigs [102]. These procedures, which are usually conducted simultaneously, are regarded within the industry as necessary to minimise problems caused by intensive production systems, such as large litters, high stocking density, successive social mixtures, together with lack of contact with soil and barren environments.

Teeth clipping is the removal of canine teeth using pliers or other sharp objects. This practice, which is conducted to minimise biting injuries to the sow’s udder or possible fights over teat disputes between littermates [103], is extremely painful [5]. Teeth clipping causes oral lesions in piglets due to tooth fragments and the exposure of the dental pulp, predisposing to gingivitis [103]. An alternative to this practice is the abrasive grinding of the sharp end of the teeth using an electric whetstone grinder. Teeth clipping and wearing increase the piglets’ cortisol levels [102] and can be a gateway to neonatal infections [103]. Although the bites caused by piglets’ teeth are harmful, some studies suggest that both practices are also harmful and stressful to the piglets and can result in lower weight gain in early lactation [71].

Tail docking, which is intended to control tail biting, can be conducted with sharp objects or cauterizers and is usually performed without the aid of analgesia or anaesthesia. Tail docking itself is painful [104], and, additionally, pigs with an amputated tail may experience pain that resembles neuropathic pain reported in humans, i.e., pigs experience persistent pain on the incision site long after the tail tissue has healed [105]. It has also been shown that besides acute pain and stress, tail docking may have adverse consequences on human–animal relationships via a fear response [104].

Male piglets are usually castrated in the first week of life to eliminate boar taint in the meat of slaughtered pigs, caused by the volatile substances androstenone and skatole that accumulate in male pig fat [106]. Surgical castration is routinely performed without pain relief. Pigs’ castration is conducted cutting the skin, exposing and breaking the spermatic cord [4]. Circulating cortisol levels increase immediately after the procedure, possibly as a result of intense pain, added to the stressful handling of containment [4,5]. In addition, castration is a risk factor for infections and for AMU in weaned piglets [72].

Sows feel pain during parturition, which can persist for up to 24 h after the birth of the last piglet [107] but awareness towards the issue is only recent and therefore interventions are rare. Providing analgesic medication for dystocic sows can reduce sows’ suffering and improve piglet immune competence. For example, oral meloxicam administered orally to sows at the beginning of farrowing increased the concentration of IgG in piglets’ serum [108] and the concentration of immunoglobulins and cytokines in the colostrum of medicated sows [109].

#### 3.2.4. Mixing Unfamiliar Animals

In intensive farms, pigs often are repeatedly mixed, starting during lactation when cross-fostering may be used, at weaning, during fattening, and after each cycle in group gestation systems. At weaning, piglets are typically separated by weight, a routine farm practice that aims to reduce fights over food that could negatively impact growth. Fattening pigs are mixed when they are moved to new housing for fattening or when they are transported to new farms. Gilts are mixed when moved to the breeding group and sows after weaning and insemination and, in some cases, after the first few weeks of gestation in crates [46]. Mixing unfamiliar animals generate fights related to the establishment of a social hierarchy, which is exacerbated by the stress associated with the novelty [97]. Management factors, such as sex, the weight of the animals, and the type of social mixtures, interfere in the occurrence of fights [110].

Aggressive interactions are already observed in nursing piglets, but after the teat order is established, fights and bites tend to decrease. Throughout the growing and fattening period, the fights are frequent, especially involving males [111]. Aggressive behaviours may also be frequent in group-housed gestating sows, often related to the management of dynamic groups, resource availability and access, and disputes over resting areas; fighting occurs when a new group of pregnant sows is housed, and the frequency declines as the social hierarchy are established [46,112]. Increased fighting and aggressiveness within a group of sows may be observed when food is not supplied simultaneously to all the sows [61].

Agonistic interactions resulting from social mixtures can impair the immune response of post-vaccination piglets, especially in castrated males, possibly because the stress of fights is associated with the suffering of castration [31]. Additionally, the HPA axis is activated in response to aggressive agonistic interactions, resulting in increased cortisol and impaired immune response [111]. Finally, skin lesions resulting from aggressive interaction predispose pigs to infections and AMU [46].

#### 3.2.5. Human–Animal Interactions and Fear

The quality of human–animal interactions should be included as one of the predictors of quality of life for pigs. Although it is difficult to argue for a direct relationship between negative human–animal interactions and diseases or AMU, the relationship with fear and physiological stress is well documented in pigs and many other species [29]. Pigs and humans communicate through acoustic, visual, tactile, and chemical sensory cues [113], and it has been shown that animals have the ability to recognise and remember aversive handlers and respond accordingly [29]. Although many routine interactions may appear innocuous, aversive human contacts that trigger a fear response in animals occur in routine pig management [29]. Additionally, humans are present throughout stressful and painful procedures applied in intensive pig farming; therefore, the quality of the interactions may either exacerbate or minimise the physiological responses reviewed earlier, which challenge homeostasis and can be a risk to the health and welfare of pigs [113].

## 4. So, Is There a Relationship between Animal Welfare and Use of Antibiotics in Pig Farming?

We have discussed several housing and management factors that are a source of distress in pigs and that ultimately facilitate the occurrence of diseases, which are commonly prevented or treated with antibiotics. Unfortunately, to date, there is a scarcity of scientific literature reporting a direct causal relationship between distress (i.e., poor welfare) in pigs and AMU. However, the examples evidenced in this review are a relevant starting point for the discussion of the relationship between animal welfare, disease, and AMU in pig production. Additionally, growing evidence is provided by studies showing that when management is focused on adopting biosecurity practices, it is possible to reduce AMU in pig farms (e.g., [114,115,116,117].

An important reason for AMU in pig farms is the prevention or treatment of respiratory, enteric, and reproductive diseases [50,93] that are facilitated by several housing and management stressors reviewed here. We have also summarised evidence that pigs’ early life concentrates a great deal on highly stressful management practices, while others have shown that this period coincides with high AMU [50,92,118]. For example, even in Germany, where antibiotics are not used for prophylactic treatments, piglets were found to be by far the most commonly treated age group [93]. Other studies have reported that farmers often use strategic oral treatment of all pigs with antibiotics around stressful managements highlighted in this review, such as castration, weaning, and at the start of the finishing period when pigs are exposed to novel physical and social environments [50,90,91].

Additionally, we reviewed the evidence of an association between AMU and specific housing and management stressors or animal factors on pig farms. For example, cross-fostering and piglet low body weight or poor weight gain were associated with AMU [72]. Poor air quality and poor cleanliness combined with poor conditions of facilities were associated with increased AMU for respiratory diseases; inadequate drinking equipment, lack of enrichment, and a poor condition of pens combined with high stocking density were associated with AMU for joint infections; and inadequate stocking density and poor enrichment were associated with AMU for tail biting [38]. Supporting the role of many housing and management factors identified in this review as important stressors for pigs, a study showed that farms with lower AMU levels were those using access to outdoors and to roughage, natural ventilation, bedding, lower stocking densities, and later weaning [119].

Lastly, it is important to bear in mind that although biosecurity and the quality of management are major risk factors for AMU [38,92,120] and AMR [121] on pig farms, they are a part of the puzzle of the current AMU challenges in the pig industry. However, good health through disease prevention is just one aspect of animal welfare. Our review has highlighted that to reduce AMU, we need to strive for happier pigs, i.e., pigs who are not stressed, bored, or fighting for a place in the group; pigs that are not left behind due to lack of space or resources. The question is how can we achieve these happier pigs? Below, we summarise evidence of how this could be achieved to some extent and thus improve pig welfare in commercial farms.

## 5. Improving Housing Environment and Management Practices to Reduce Stress in Pig Farming

During the past decades, many changes in housing and management that may minimise or eliminate the environmental stressors described above have been proposed, tested, and even implemented with success in commercial farms [41]. Such housing and management changes, together with the elimination of painful procedures or their replacement with less invasive alternatives, have proven to allow the expression of species-specific behaviours, reduce stress, boost the immunological system, and even promote positive emotional states [41,122]. In this section, we present an overview of known strategies focusing on the gains attained through environmental enrichment (including better feeding practices and on-farm resources organisation), reduction in stocking density, group-housing and family rearing, increased age at weaning, neonatal socialisation, the prevention and treatment of lameness, automatic feeding, and supply of fibre for sows.

### 5.1. Improved Housing and Environmental Enrichment

A key overarching solution to the distress and boredom suffered by pigs is good housing design and environmental enrichment of their living space. Good housing is a design that enables pigs to move, explore, and feel protected from threats and that ensures thermal comfort [41,123]. The provision of hiding opportunities is an area often forgotten in commercial farms; however, it can protect newly housed sows from aggressive interactions with resident sows [124], and building barriers and hiding places can also be an alternative to reduce agonistic interactions and stress [125]. On the other hand, the provision of space for locomotion is beneficial for the health of the locomotor system. Thus, increasing the space per sow in indoor gestation group housing can reduce the frequency of lameness [126]. Moreover, loose farrowing housing provides the additional space for sows to move and also improves maternal behaviour in sows and social behaviour in piglets; positive impacts are observed even when the sows’ movement is restricted for a few days after farrowing with the aim to reduce piglet mortality [127].

Environmental enrichment consists of actions that make the living space attractive, giving pigs the opportunity to express highly motivated innate behaviours [128]. Substantial evidence exists about the positive behavioural value enriched environments can have in pigs, including the promotion of positive affective states at all stages of rearing. Examples of enrichment include the provision of straw, toys, and manipulable materials for weaned pigs that reduce the incidence of redirected oral behaviours [129,130]. In growing pigs, the supply of manipulable materials can aid in reducing the incidence of tail biting [37,131,132]. Recent work showed that environmental enriched housing could have positive effects on the development of the immune system and the establishment of gut microbiota in early life [133]. Enabling sows to build a nest and offering straw during farrowing and lactation can prevent skin and claw lesions in piglets [134], increase activity, and reduce abnormal behaviour in the sows [135]. Furthermore, there is a knock-on effect of reducing sows’ stress at the end of gestation in that it may positively affect the offspring, influencing the activity of the HPA axis and reducing the incidence of aggressiveness and belly-nosing [136]. Additionally, fibre (i.e., provision of straw in sow diet) increases the volume and absorbs water, stimulating the mechanical receptors of the stomach and decreasing gastric emptying, necessary for satiety [60]. Thus, straw inclusion will reduce levels of ghrelin and chronic hunger sensation, as well as the incidence of gastric ulcerations and agonistic interactions between sows [60,137]. Enrichment of the maternity housing with chewable materials can stimulate the exploratory behaviour of the piglets and increase the frequency of non-painful contact of the piglets with the udder, reducing the stress and severity of skin lesions in lactating sows [138].

### 5.2. Group Housing-Increasing Space, Reducing Stocking Density or Both

Good housing should also encompass the social dynamics and stocking density for it to fulfil the goal of improving welfare. Group housing can be beneficial for pregnant and lactating sows; if well managed, it may reduce serum cortisol concentration and reduce the frequency of vacuum chewing and sitting behaviour [139,140]. Improving the housing conditions of sows can have positive effects on the health of piglets; piglets of sows reared in groups showed greater resistance and resilience when challenged with LPS compared to piglets of individually housed sows [140]. Moreover, group housing enables early socialisation among piglets reducing agonistic interactions during weaning [35]. However, group housing can fall into a success in research/unsuccessful in practice paradox if provided with insufficient space (i.e., high stocking density), poor environmental enrichment, inadequate resource allocation, or poor feeding management. Such situations create failed living spaces that increase agonistic interactions, injuries, and overall reduction in welfare [41].

### 5.3. Reducing Pre-Natal, Neonatal and Weaning Stress and Promoting Positive Human–Animal Interactions

Weaning piglets at later ages in the absence of early painful procedures (i.e., castration, tail clipping, teeth clipping) has positive impacts on piglet behaviour and GIT structure and function [73]. Additionally, later-weaned piglets with optimal feeding strategies (i.e., creep feeding) have greater diversity and abundance of bacterial microflora in the gastrointestinal tract, which may help reduce the incidence of diarrhoea and the use of antibiotics in this critical phase [73,89]. Delayed weaning by itself is not enough; maintaining the social group and the physical environment at weaning is equally important to reduce weaning stress and improve post-weaning feed intake [97]. Piglets with permanent social structures are better socialised, which improves their overall adaptability to the post-weaning environment and reduces cortisol levels, agonistic interactions, and the resulting lesions, whilst increasing play behaviour [89,141].

Eliminating painful procedures or replacing them with known alternatives (e.g., immunocastration, environmental enrichment, reduced stocking density, family systems) facilitates caregivers’ attentiveness and empathy towards animals, central for pig production systems that advocate for high animal welfare [142]. In addition, calm, gentle, and pleasurable human–animal interactions (i.e., pleasant management routines) can decrease stress, reduce behaviours that denote fear and anxiety, and keep working environments peaceful [29]. Positive human–animal interactions can include friendly human presence, contact, and tactile stimuli (scratching or caressing), providing food and objects for interaction (i.e., environmental enrichment), and engaging in peaceful and pleasant routines with the animals [143]. Prolonged gentle handling has proven effective at reducing stress and anxiety in pigs [144], as well as in pregnant and lactating sows [145] and piglets that also gained more weight [146]. Finally, positive human–animal interactions are essential to ensure the welfare and quality of life of pigs on farms and maintain the social license of the pig industry and reduce the use of antibiotics in farms.

### 5.4. Making a 180 Degree Turn into Genetic Selection to Improve Animal Welfare

Historical genetic selection has contributed to the success of intensive pig production through the development of genetic strains with highly productive characteristics, such as improved weight gain, feed conversion, and hyperprolificity. The use of this knowledge to develop animals more resilient to environmental stressors or more resistant to pathogens may contribute to the aim of reducing AMU in intensive pig production. For example, some promising studies have identified genotypes resistant to diseases such as circovirus [147] and Mycoplasma hyopneumoniae [148,149]. As noted in the discussion above, many stressors that affect pigs’ wellbeing and health are exacerbated in large litters, such as increased duration of farrowing, low birthweight piglets, and a lack of teats for all piglets. For example, a retrospective study of Swedish pig herds showed a correlation between the increase in litter size and an increased need for treatments with antibiotics in sows due to puerperal infections [150]. Thus, limiting litter size should be considered if striving for sustainability in the pig industry [151]. Genetic selection for lower aggressiveness, in the case of females, in addition to the reduction in agonistic interactions, can lead to increased maternal ability since these traits are correlated [152]. Another example is the selection for low levels of skatole, which would eliminate the need for castration, reducing fights between pigs during the fattening period without lowering the quality of meat by the presence of boar taint [153]. Gene editing technologies offer promising opportunities to introduce beneficial characteristics to commercial pig strains without competing with production traits, provided ethical and technical limitations are addressed [154].

### 5.5. The Intrinsic Value of Pigs—Re-Centring Pig Industry Values

We have carefully selected evidence that current management systems, including the production system and the quality of practices within the system, are failing to keep pigs healthy and “happy”. In parallel, such evidence highlights an industry whose values and goals are guided by high productivity and financial returns. This fragile balance is sustained through reactive management practices (e.g., cross-fostering created as a response to hyperprolific breeds) [76] or reliance on the effectiveness of prophylactic and therapeutic AMU [50]. A recent scoping review of studies evaluating alternatives to antibiotics to prevent or control disease and reduce the need for antibiotics in nursery pigs identified a majority of studies covering feed additives and vaccines and relatively few studies evaluating housing or management practices [10], revealing a lower scientific effort/interest in systemic changes and a preference for “silver bullets” to replace antibiotics. On-farm changes are necessary to push for optimal pig welfare and reduced AMU, which demands an organisational re-centring of the pig industry values. Changes must recognise the intrinsic value of the pig within the system, the connections with its welfare, human well-being, and the environment [22].

## 6. Implications and Closing Remarks

A holistic reduction in stressors and boredom in pig herds (i.e., happier pigs) is crucial to the multi-actor and multi-action puzzle of reducing current AMU in intensive pig production farms. This will simultaneously address two demands from society: improving the welfare of pigs (ethical demand) and reducing the use of antibiotics (public health demand). We reviewed many husbandry practices used in intensive systems that challenge homeostasis, increasing the animals’ susceptibility to diseases. Examples centred around factors inherent to the housing environment (and its management), animal management, and the animal selection or genetic makeup. Likewise, we pointed out alternative actions to reduce and prevent stress. Although in some cases more applied research is needed, we have enough collective knowledge to act and seize the moment. Thus, it begs the question, why has the pig industry not done so? Post-war industrialisation of pig production was driven by increased productivity and cost reductions [155]. Although, arguably, initial industry changes developed holistically and sided with AMU [155,156,157], this review has highlighted current painful/stressful practices that are a result of the pig industry forgetting its holistic principles of production and that the transaction point is the animal, to the point of surpassing the balance of ethical and biological demands of these animals. Thus, today’s pig production is not sided with AMU; instead, it relies on using precious molecules (antibiotics) as palliative treatments to support such unsustainable production systems [50,157].

Welfare is a complex balance of different aspects of the life of animals and how they perceive/feel them. We have argued that there is indirect evidence that happier and less stressed pigs are more immune-competent, more capable of naturally defending themselves from environmental pathogens, and therefore less dependent on preventive antibiotics. The adoption of good management practices that consider the characteristics and needs of the pigs holistically is essential to meet the international call for prudent use of antibiotics. Additionally, public opinion must be recognised as a vital force for change in production systems. There is a growing literature showing that restrictive, barren housing systems and painful and stressful managements have no societal support. In contrast, alternatives focused on free systems and naturalness are not just preferred but expected by consumers [158,159,160].

A common understanding of the meaning of farm animal welfare is a necessary step to change to a production system that attends to these expectations. Yet, industry stakeholders define animal welfare in terms of biological function concerning production [87], thus minimising the importance of naturalness and affective states [3,42,142]. Thus, “good welfare” is seen as animals not falling sick or not dying on-farm before they reach their weight to slaughter and for sows that wean as many piglets as biologically possible. In turn, the current use of antibiotics is justified on the grounds that it protects the “welfare” of the animals. We agree that an animal’s health is an essential dimension that defines its welfare. Yet, there is a danger in reducing the welfare of an animal into not being sick. This reductionist approach fails to question how we got there and if there is another way to allow an animal to live happily, not just survive its environment. Thus, the lack of recognition of animal welfare in all its dimensions makes it more difficult to believe that many interventions suggested in Section 5 may improve pigs’ quality of life and make the industry less dependent on antibiotics.

Farmers are seen as the ultimate people responsible for changing the current AMU. A simplistic and polarised portrait of why pig farmers use antibiotics is that of a neglectful or a protective individual of the welfare of their animals. Today, many farmers report feeling/being powerless to think differently, and if they do, act differently, quoting that economic constraints, production standards or technical advice do not leave room for change [87,161]. Just as the pigs in their farms, many farmers are surviving the system. Thus, investments needed for any changes must be supported by the industry, consumers, and governments. A call for pig farmers to rationally reduce AMU will succeed or fail pending such external support and structural changes in the network that currently uses antibiotics as a structural material for production, at local, national, and international levels. This means that individual behaviour changes are not enough nor sustainable in the long run. Ultimately, we urgently call for a re-centring of the industry objectives (inclusive of all stakeholders) into the intrinsic values of life (a life worth living) and nature (a place worth living) for all living creatures. The solutions demand leaving behind the conception that feeding the world means intensifying animal production, towards a genuinely sustainable approach where keeping our world means slowing down production.

## Data Availability

Not applicable.
